# GSK-3β regulates tumor growth and angiogenesis in human glioma cells

**DOI:** 10.18632/oncotarget.5043

**Published:** 2015-09-10

**Authors:** Peng Zhao, Qi Li, Zhumei Shi, Charlie Li, Lin Wang, Xue Liu, Chengfei Jiang, Xu Qian, Yongping You, Ning Liu, Ling-Zhi Liu, Lianshu Ding, Bing-Hua Jiang

**Affiliations:** ^1^ Department of Neurosurgery, The First Affiliated Hospital of Nanjing Medical University, Nanjing 210029, China; ^2^ State Key Lab of Reproductive Medicine, Department of Pathology, and Collaborative Innovation Center for Cancer Personalized Medicine, Cancer Center, Nanjing Medical University, Nanjing 210029, China; ^3^ Department of Neurosurgery, Huai'an First People's Hospital, Nanjing Medical University, Huai'an 223300, China; ^4^ Department of Pathology, Nanjing Drum Tower Hospital, The Affiliated Hospital of Nanjing, University Medical School, Nanjing 210008, China; ^5^ Department of Environmental Toxicology, University of California-Davis, Davis, CA 94564, USA; ^6^ Ninggao Personalized Medicine and Technology Innovation Center, Nanjing 21130, China; ^7^ Department of Pathology, Anatomy and Cell Biology, Thomas Jefferson University, Philadelphia, PA 19107, USA

**Keywords:** GSK-3β, glioma, tumor growth, angiogenesis, mTOR

## Abstract

**Background:**

Glioma accounts for the majority of primary malignant brain tumors in adults.

**Methods:**

Glioma specimens and normal brain tissues were analyzed for the expression levels of GSK-3β and p-GSK-3β (Ser9) by tissue microarray analysis (TMA) and Western blotting. Glioma cells over-expressing GSK-3β were used to analyze biological functions both *in vitro* and *in vivo*.

**Results:**

The levels of p-GSK-3β (Ser9), but not total GSK-3β, are significantly up-regulated in glioma tissues compared to normal tissues, and are significantly correlated with the glioma grades. Ectopic expression of GSK-3β decreased the phosphorylation levels of mTOR and p70S6K1; and inhibited β-catenin, HIF-1α and VEGF expression. Forced expression of GSK-3β in glioma cells significantly inhibited both tumor growth and angiogenesis *in vivo*.

**Conclusions:**

These results reveal that GSK-3β regulates mTOR/p70S6K1 signaling pathway and inhibits glioma progression *in vivo*; its inactivation via p-GSK-3β (Ser9) is associated with glioma development, which is new mechanism that may be helpful in developing GSK-3β-based treatment of glioma in the future.

## INTRODUCTION

Gliomas are the most aggressive types of primary intracranial tumors and constitute almost 80% of primary brain tumors. In the last 5 years, despite with the improvement of some treatments, such as aggressive surgery, radiation and chemotherapies, the median survival (MS) rate of patients with malignant glioma only improved from 10 to 14 months after diagnosis [1, 2]. One of the special characters of glioma is well-perfused blood vessel with very high density in the brain, which is different from many other types of cancers. Angiogenesis is the formation of new blood vessels out of the pre-existing vasculature, and is vital for tumorigenesis and tumor development [3]. Glioma usually manifests itself as a focal lesion with central necrosis surrounded by an angiogenic tumor rim (one of the characteristics of GBM); this type of tumor invades the surrounding extracellular matrix, using both white matter tracts and blood vessels as substrates. Extensive studies have confirmed that angiogenesis is required for malignant glioma growth [4, 5].

GSK-3 is a multifunctional Ser/Thr kinase, which was first identified as a critical mediator in glycogen metabolism and insulin signaling [6, 7]. It is now known that GSK-3 is an important component of diverse signaling pathways involved in the regulation of protein synthesis, glycogen metabolism, cell mobility, proliferation and survival [8, 9]. There are two mammalian GSK-3 isoforms encoded by distinct genes: GSK-3a and GSK-3β, which share 85% identity [10]. Despite a high degree of similarity and functional overlap, these isoforms are not functionally identical and redundant. It is now known that GSK-3β plays a central role in a variety of signaling pathways, such as the Wnt/β-catenin, Hedgehog, Notch and insulin signaling pathways [11–15]. Accordingly, GSK-3β activity depends on the balance of phosphorylation levels at activating Tyr216 site and inactivating Ser9 site levels. A number of kinases can phosphorylate its N-terminal serine-9 residue, including AKT and mitogen-activated protein kinase (MAPK)-1, leading to the auto-inhibition of GSK-3β. The dysregulation of GSK-3β has also been implicated in tumorigenesis and cancer progression [8, 11, 16–18]. However, the role of GSK-3β and GSK-3β Ser9 phosphorylation in glioma development is unclear, and the mechanisms underlying GSK-3β regulation of neoplastic transformation and tumor development remains to be elucidated.

In this study, we applied tissue microarray technique to detect a significant increase of p-GSK3β-Ser-9 levels in most glioma tissues, which is associated with patients with high-grade gliomas and metastatic tumors. Moreover, overexpression of GSK-3β inhibited angiogenesis and tumor growth *in vivo*. These studies elucidate the specific role of GSK-3β in glioma, and helped further determine the underlying mechanism of GSK-3β in regulating glioma development.

## RESULTS

### Increase of GSK-3β phosphorylation levels at Serine 9 in glioma tissues

**Figure 1:**
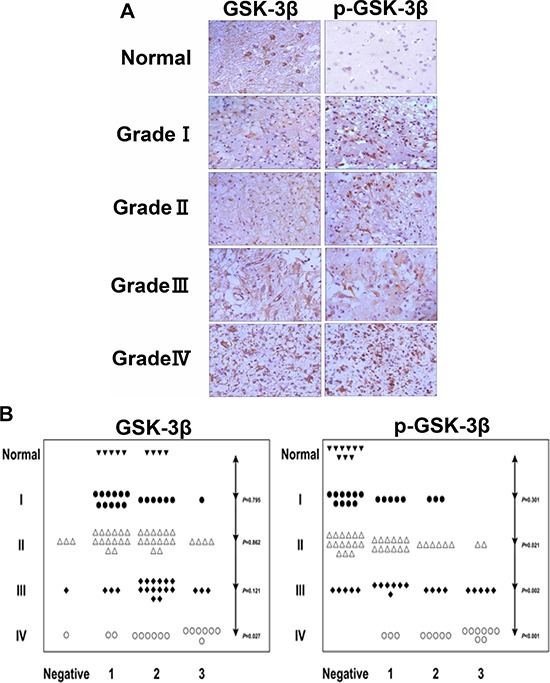

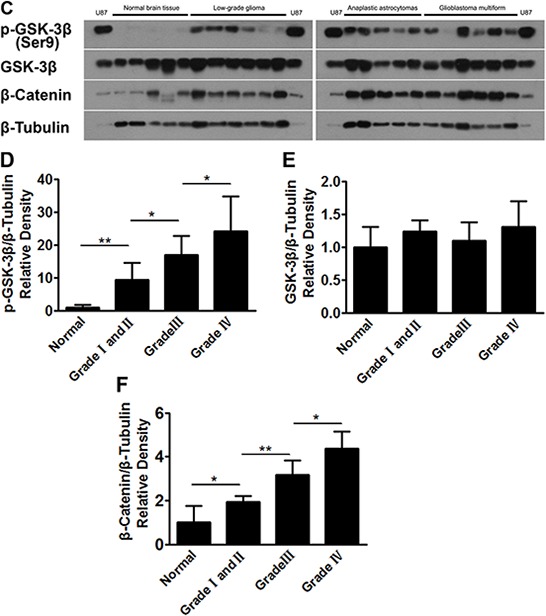
Human glioma tissues express high levels of p-GSK-3β (Ser9) **A.** The expression levels of GSK-3β and p-GSK-3β (Ser9) in normal brain tissues and four (I, II, III, IV) grades of glioma tissues were analyzed by human tissue mircroarry, bar 40 μm. **B.** Relative expression levels of GSK-3β and p-GSK-3β (Ser9) in human normal brain tissues and four grades of glioma tissues were analyzed by two experienced pathologists in a blind manner and marked as final IHC scores; *P* value represents the difference of GSK-3β and p-GSK-3β (Ser9) expression levels between normal brain tissues and four grades of gliomas sections. **C.** Levels of GSK-3β, p-GSK-3β (Ser9) and β-catenin in human normal brain tissues and four grade gliomas tissues were determined by Western blotting; 20 μg of protein extracts from U87 cells was used, and β-tubulin was used as an internal loading control. **D, E.** and **F.** Relative densities were quantified using ImageJ software. Results are presented as mean ± SD from three duplicates. Asterisk indicates significant difference when compared to the control (*P* < 0.05).

The expression levels of GSK-3β and p-GSK-3β (Ser9) in 9 normal tissue specimens and 90 glioma tissues of paraffin-embedded formalin-fixed were detected by Tissue Microarray Analysis (TMA). The results showed that the expression levels of p-GSK-3β (Ser9) are significantly up-regulated in glioma tissues compared with normal tissues (Figure 1A). The relative expression levels of GSK-3β and p-GSK-3β (Ser9) were analyzed by two experienced pathologists in a blind manner and marked as final IHC scores: 0 (negative), + (weak), ++ (moderate) and +++ (strong). Types of tissues used are listed in Table 1. Higher p-GSK-3β (Ser9) expression levels were detected in 60 (67%) of 90 glioma cases, and were significantly correlated with higher glioma grade (Figure 1B). There was no significant correlation between total expression levels of GSK-3β with the glioma grade and normal tissue specimens (Figure 1B). We further analyzed the expression levels of GSK-3β and p-GSK-3β (Ser9) in 5 normal brain tissues and 33 glioma specimens by Western blotting assay. Pathologic features of tissue samples are listed in Table 2. Consistent with the results in TMA, the expression levels of p-GSK-3β (Ser9), but not total GSK-3β, were greatly increased in glioma tissues, and were positively correlated with higher glioma grades (Figure 1C, and 1E). Since GSK-3β is the initiation step of proteasome-dependent degradation of β-catenin, we also examined levels of β-catenin in glioma tissues. As shown in Figure 1C and 1F, the levels of β-catenin expression in glioma tissues were significantly increased compared with normal tissues, and higher grades of glioma are associated with higher expression levels of β-catenin. These results indicated that p-GSK-3β (Ser9) plays an important role in increasing β-catenin expression and is a potential biomarker of glioma development.

**Table 1 and 2:** The information of human brain tissues (types and WHO criteria) was summarized in Table I and II

I		
Type of tissues	WHO grade	Number of cases
Normal brain tissue		9
Pilocytic astrocytoma	I	18
Diffuse astrocytoma	II	9
Oligoastrocytoma	II	15
Oligodendroglioma	II	11
Anaplastic gliomas		21
Anaplastic astrocytoma	III	7
Anaplastic oligoastrocytoma	III	7
Anaplastic oligodendroglioma	III	7
Glioblastoma multiforme	IV	16
II		
Type of tissues	WHO grade	Number of cases
Normal brain tissue		5
Low-grade gliomas		6
Pilocytic astrocytoma	I	2
Diffuse astrocytoma	II	1
Oligoastrocytoma	II	2
Oligodendroglioma	II	1
Anaplastic gliomas		5
Anaplastic astrocytoma	III	2
Anaplastic oligoastrocytoma	III	2
Anaplastic oligodendroglioma	III	1
Glioblastoma multiforme	IV	6

### GSK-3β inhibits β-catenin and mTOR/p70S6K1 signal pathways, and down-regulates HIF-1 and VEGF expression

**Figure 2:**
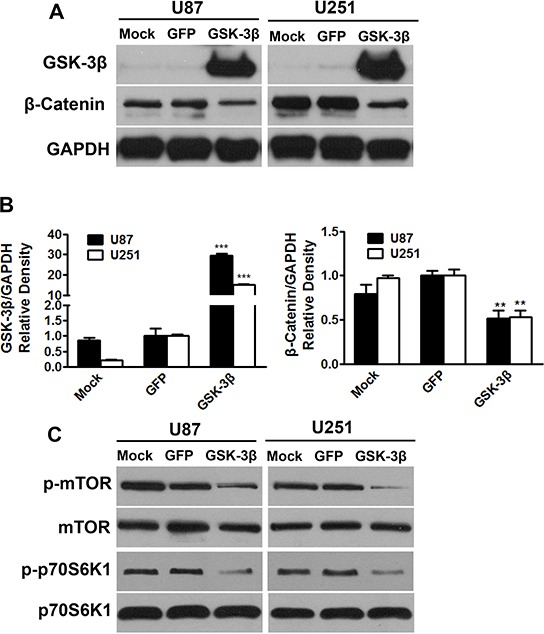

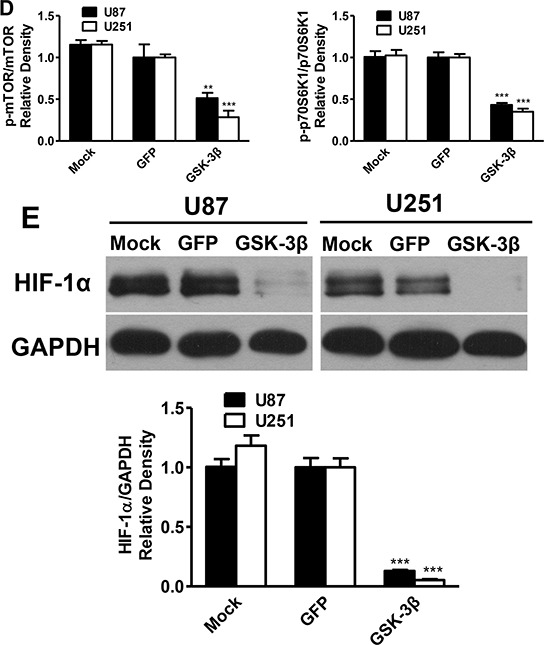

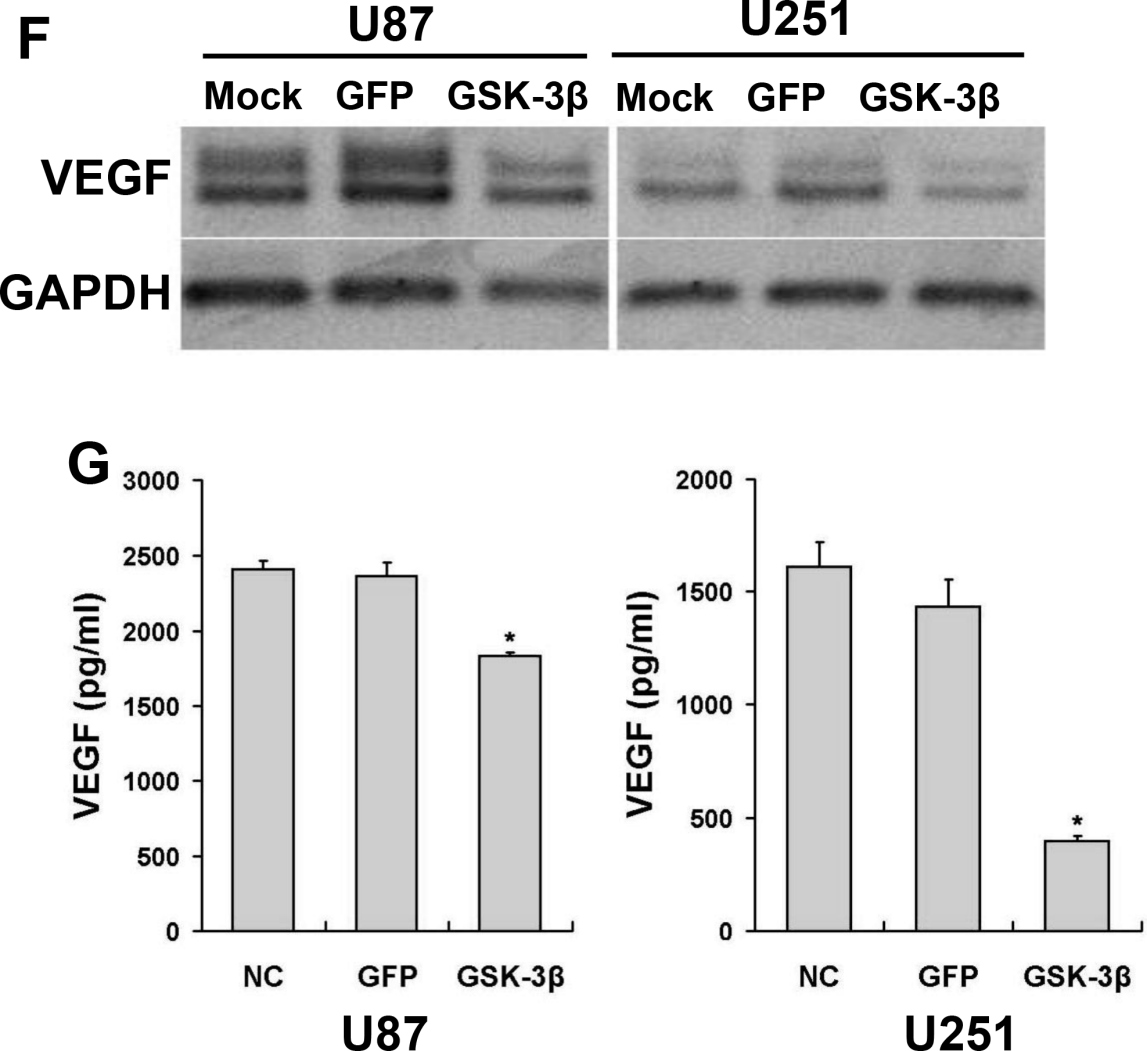
Overexpression of GSK-3β attenuated β-catenin and mTOR/p70S6K1 activation in human glioma cells **A.** U87 and U251 cells were transfected with or without adenovirus carrying GSK-3β or GFP at 10 MOI. After transfection for 48 h, the expression levels of GSK-3β, β-catenin and GAPDH were detected by immunoblotting. **B.** Relative densities were quantified using ImageJ software. Asterisk indicates significant difference when compared to the GFP group (*P* < 0.05). **C.** Cells transfected with or without adenovirus carrying GSK-3β or GFP were subjected to Western blotting analysis for mTOR, p-mTOR, p70S6K1, p-p70S6K1, HIF-1α and GAPDH expression. The protein levels from three independent experiments are quantified and presented as mean ± SD. Asterisk indicates significant difference when compared to the control (*P* < 0.05). **D.** and **E.** Cells transfected with or without adenovirus carrying GSK-3β or GFP were subjected to Western blotting analysis for mTOR, p-mTOR, p70S6K1, p-p70S6K1, HIF-1α and GAPDH expression. The protein levels from three independent experiments are quantified and presented as mean ± SD. Asterisk indicates significant difference when compared to the control (*P* < 0.05). **F.** and **G.** VEGF mRNA and protein levels were detected by semi-quantitative RT-PCR assay and ELISA assay, respectively. Asterisks indicate significant difference when compared to the control (*P* < 0.01).

To understand the mechanism of GSK-3β in regulating glioma development, human glioma cell lines U87 and U251 were infected with the adenovirus carrying GSK-3β cDNA (Ad-GSK-3β) or GFP control (Ad-GFP). The expression levels of GSK-3β were analyzed in these cell lines U87-GFP, U87-GSK-3β, U251-GFP, U251-GSK-3β by Western blotting. As shown in Figure 2A and 2B, expression levels of GSK-3β were similar in mock cells and GFP cells, whereas GSK-3β levels were greatly increased in GSK-3β-overexpressing U87 and U251 cells. This result demonstrated that these adenovirus infected cells successfully overexpress GSK-3β. To identify signaling pathways that may be regulated by GSK-3β, we demonstrate that delivery of exogenous GSK-3β attenuated the expression levels of β-catenin (Figure 2A and 2B) and decreased the phosphorylation levels of mTOR and p70S6K1 (Figure 2C and 2D). No significant differences were found in the levels of total mTOR and p70S6K1 in any of the cells. MTOR/p70S6K1 signaling pathway is known to be involved in regulation of HIF-1α stability [21]. Overexpression of GSK-3β is sufficient to decrease HIF-1α level (Figure 2E). It was reported that VEGF is regulated by HIF-1α through hypoxia responsive element [22]. Here we demonstrate that GSK-3β inhibits VEGF mRNA and protein levels by RT-PCR and ELISA assay (Figure 2F and 2G). These results showed that ectopic expression of GSK-3β decreased the phosphorylation of mTOR and p70S6K1; and inhibited β-catenin, HIF-1α and VEGF expression.

### GSK-3β inhibits tumor angiogenesis *in vivo*

**Figure 3:**
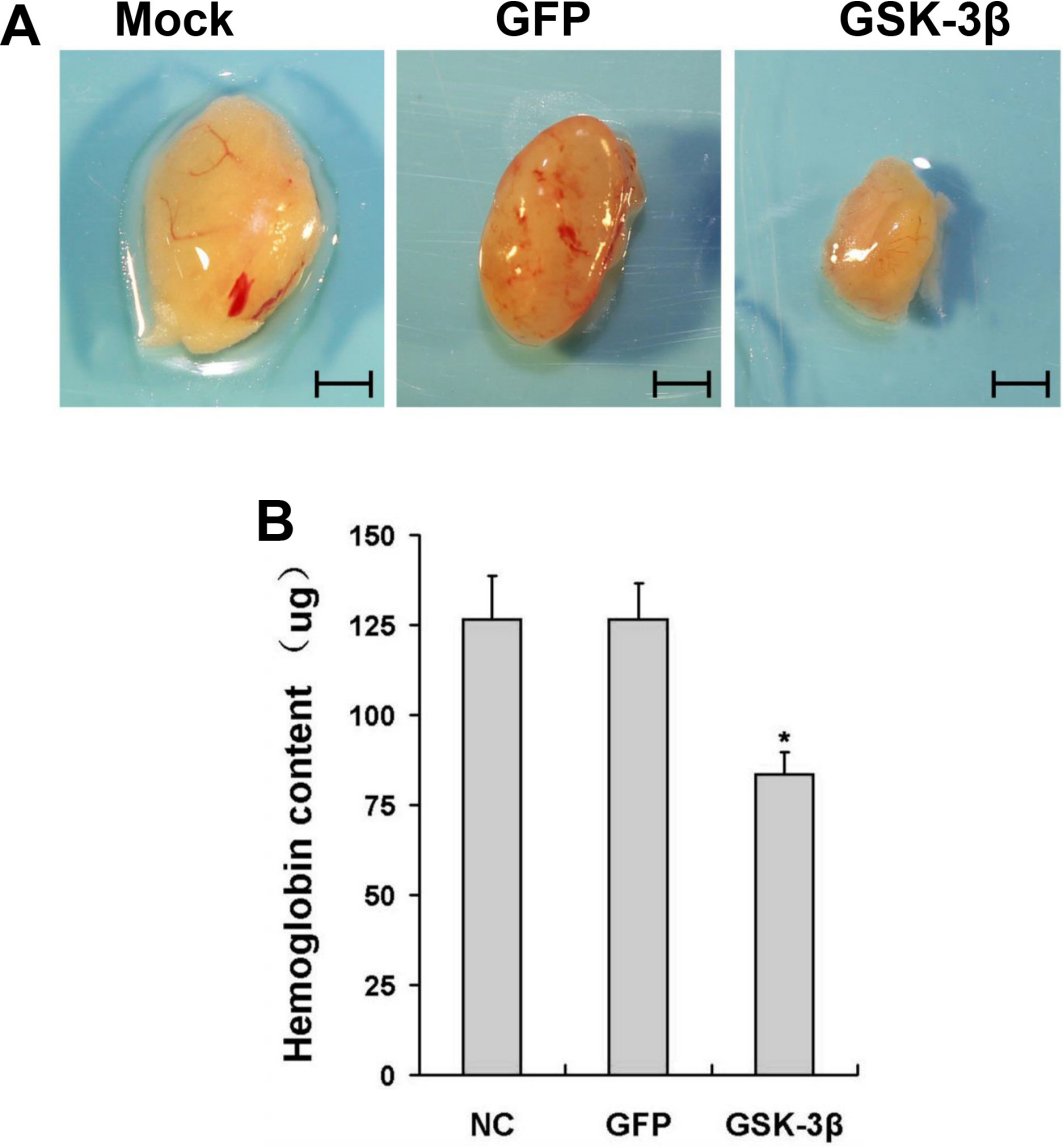
Overexpression of GSK-3β inhibited tumor angiogenesis *in vivo* **A.** U87 cells were transfected with or without adenovirus carrying GSK-3β or GFP at 10 MOI. After the cells were cultured for 48 h, aliquots of cells (1.0 × 10^6^ cells, 0.1ml) were mixed in 1:2 ratio with growth factor-reduced phenol red-free Matrigel, and injected subcutaneously into both flanks of nude mice. After 12 days of implantation, the representative plugs from each group were shown. Scale bar: 5 mm. **B.** The hemoglobin levels in plugs (*n* = 10). * indicates significant difference when compared to that of the GFP group (*P* < 0.05).

HIF-1α/VEGF pathway is a major angiogenesis signaling pathway in both physiological and pathological processes [23]. To examine the potential roles of GSK-3β in angiogenesis, we performed angiogenesis assay *in vivo*. U87-GSK-3β cells were trapped in growth factor-free Matrigel, and implanted into both flanks of nude mice. Uninfected and U87-GFP cells were used as the negative control. U87 cells induced angiogenesis, which was inhibited by GSK-3β-overexpression (Figure 3A). The hemoglobin levels in the plugs were used to evaluate angiogenesis responses. Ectopic expression of GSK-3β decreased hemoglobin levels by 35% compared to the GFP group (Figure 3B). These results demonstrate that GSK-3β inhibit tumor-induced angiogenesis.

### GSK-3β expression suppresses tumor growth *in vivo*

**Figure 4:**
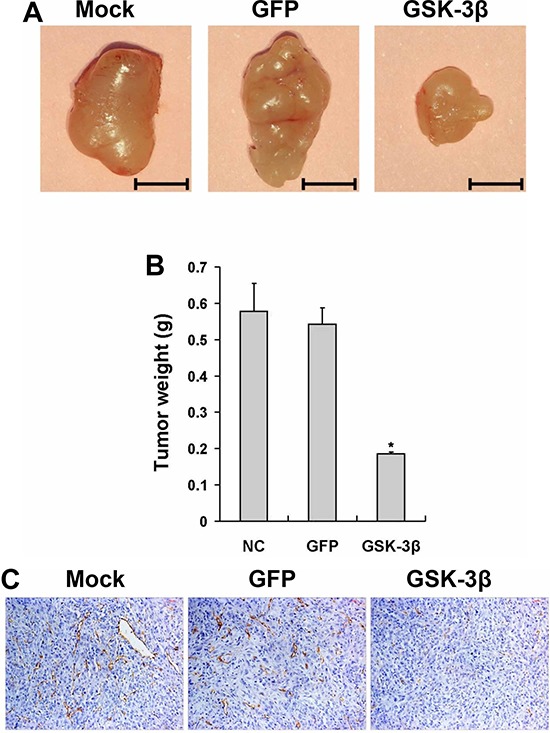

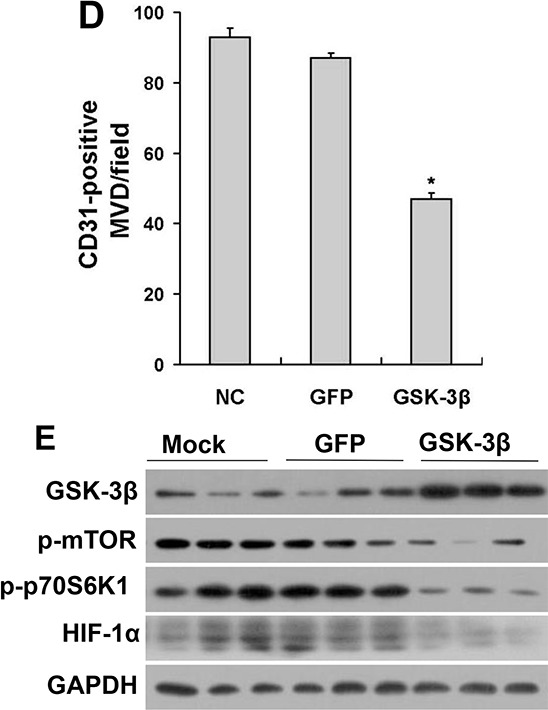

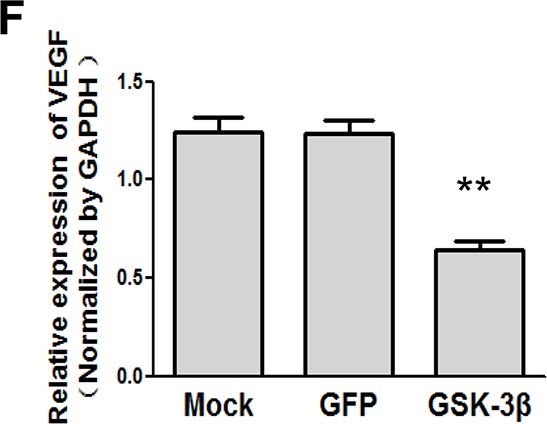
Overexpression of GSK-3β decreased tumor growth *in vivo* **A.** U87 cells were transfected with or without adenovirus carrying GSK-3β or GFP at 20 MOI. After the cells were cultured for 48 h, 3 × 106 cells were injected into both flanks of nude mice. Representative tumors 35 days after implantation from each group were shown. (Bar: 5 mm) **B.** The tumor weight was measured for each xenograft. Asterisks indicate significant difference when compared to the GFP group (*P* < 0.05). **C.** Representative image of CD31-stained sections. **D.** Quantitative analysis of MVD in the xenograft plugs sections. The graph represents the mean ± SD from five different tumor sections for each group. *indicated significant difference at *p* < 0.05. **E.** The protein levels of mTOR, p-mTOR, p70S6K1, p-p70S6K1 and HIF-1α in tumor tissues were analyzed by Western blotting. GAPDH was used as internal control. **F.** The mRNA levels of VEGF were analyzed in tumor tissues by semi-quantitative RT-PCR assay.

Angiogenesis is required for tumor growth and metastasis. To further determine whether GSK-3β activation in U87 is sufficient in inhibiting tumor growth, we generated xenograft tumors by the injection of U87-GSK-3β or U87-GFP control cells subcutaneously into nude mice for 35 days. As shown in Figure 4A, the sizes of tumors from GSK-3β-expressing cells were much smaller than those from the GFP control group after 5 weeks of the injection. GSK-3β overexpression decreased tumor growth by 65% compared to the Ad-GFP group (Figure 4B). Then, representative sections of the tumors were stained using monoclonal antibody against CD31. Consistent with the results in angiogenesis assay, the immunohistochemical analysis indicated that there were a rare numbers of CD31-positive microvessels from GSK-3β-expressing group compared with GFP control group, revealing that GSK-3β overexpression significantly reduced the formation of tumor microvessels (Figure 4C and 4D). We also analyzed GSK-3β, p-mTOR, p-p70S6K1 and HIF-1α expression levels by Western blotting in xenograft tumors from three groups. As expected, the higher levels of GSK-3β expression in the xenograft tumors from U87-GSK-3β cells were correlated with the lower expression levels of p-mTOR, p-p70S6K1, and HIF-1α (Figure 4E). Similarly, it was confirmed that VEGF mRNA expression in xenograft tumors were significantly decreased by GSK-3β overexpression (Figure 4F). In summary, these results demonstrate that GSK-3β overexpression inhibits tumor growth.

## DISCUSSION

Gliomas are the most common primary brain tumor. Despite aggressive treatment approaches, glioma is still hardly curable [1, 2]. Extensive studies have demonstrated that human glioma exhibits aberrant expression profiles of Wnt/beta-catenin signaling [24, 25]. GSK-3β is an important molecule to regulate Wnt/beta-catenin signaling, and is a key regulator of tumor development [26, 27]. For example, GSK-3β has been shown to regulate the JNK pathway, and its inhibition triggers apoptosis in pancreatic cancer [16]. In bladder cancer, GSK-3β positively regulates cell proliferation and survival *in vitro* [28]. To the present, a large number of studies demonstrated GSK-3β as a potential therapeutic target in ovarian, breast cancer and bladder cancer [29, 30]. However, the role and the molecular mechanism of GSK-3β in glioma still remain to be elucidated.

In order to get an insight into the biological relevance of GSK-3β in glioma, we did immunohistochemical stainings and Western blotting analysis of total GSK-3β and phospho-GSK3β at Ser-9 levels in glioma tissues. Our study shows that there is a strong expression of total GSK-3β in the vast majority of normal and glioma tissues. However, the expression levels of p-GSK-3β (Ser9) were much higher in glioma tissues when compared to normal tissues, and were correlated with higher glioma grades. These results suggest that the expression levels of phospho-GSK-3β-Ser-9 may serve as a valuable prognostic marker in glioma. To explore the functional relevance of GSK-3β in glioma, we utilized U87 cells infected with adenovirues carrying GSK-3β (Ad-GSK-3β). The functional analysis reveals that ectopic expression of GSK-3β suppressed angiogenesis and tumor growth *in vivo*.

It is reported that GSK3β is able to phosphorylate β-catenin, thus marking the protein for degradation [27, 31]. The expression levels of β-catenin were higher in glioma tissues compared to the normal tissues, and were decreased following ectopic expression of GSK-3β in U87 and U251 cells. Therefore, the Wnt/β-catenin signaling pathway may be involved in the process of glioma development. Moreover, in the present study, changes in mTOR/p70S6K1 signaling pathway occurred in U87 and U251 glioma cells following GSK-3β overexpression, suggesting that this signaling pathway may play an important role in the GSK-3β-inhibited glioma development events. It is currently unknown whether there is cross-talk between the β-catenin and mTOR cascades in this regard or if they work independently to strengthen development. Further investigations are needed to reveal resolve the relationship between these two signaling pathways in the context of GSK-3β-inhibited glioma development.

The mTOR/p70S6k1 signaling pathway is known to be involved in regulation of HIF-1α stability [32]. VEGF is a key proangiogenic activator which can be induced at transcriptional level by HIF-1α, and well known to play an important role in tumor growth and angiogenesis. Our previous studies revealed that high expression of VEGF defines carcinomas with more tumor growth and angiogenesis and poor prognosis. In this study, overexpression of GSK-3β dramatically down regulates HIF-1α protein expression as well as VEGF mRNA levels, Thus, HIF-1α/VEGF pathway may be involved in transmitting the biological effects of GSK-3β- inhibited tumor growth and angiogenesis.

In conclusion, our current study demonstrates that p-GSK-3β (Ser9) levels are positively related with higher glioma grades; ectopic expression of GSK-3β is sufficient to inhibit angiogenesis and tumor growth; we further identify Wnt/β-catenin and mTOR/p70S6K1/HIF-1a/VEGF pathways are involved in GSK-3β-inhibited angiogenesis and tumor growth. Taken together, these findings suggest that p-GSK-3β (Ser9) may be a useful biomarker for glioma development, and provide new information for using GSK-3β-based diagnostics and/or potential therapeutic target for glioma treatments in the future.

## MATERIALS AND METHODS

### Cell culture and reagents

Human glioma cell lines U87 and U251 (American Type Culture Collection, Manassas, VA, USA) were cultured in DMEM supplemented with 2 mmol/L l-glutamine, 10% fetal bovine serum, 100 units/mL penicillin, and 100 mg/mL streptomycin, 5% CO2 at 37°C. Antibodies against GSK-3β, phospho-GSK-3β (Ser9), β-catenin, AKT, phospho-AKT (Ser473), p70S6K1, phospho-p70S6K1, mTOR, phospho-mTOR, β-Tubulin were obtained from Cell Signaling (Beverly, MA, USA). HIF-1α was from BD Biosciences (Franklin Lakes, NJ, USA), and GAPDH was purchased from KangCheng Biotech (Shanghai, China), while antibody against CD31 was supplied by Abcam (Cambridge, UK).

### Tissue samples

Tissues were obtained from the first affiliated hospital of Nanjing Medical University, Nanjing, China. Total 9 human normal brain tissues and 90 human glioma tissues were obtained as paraffin-embedded formalin-fixed blocks. Fresh 5 normal brain tissues and 33 glioma specimens were collected immediately after the surgical removal, and snap-frozen in liquid nitrogen. All patients were provided written informed consent, and the experimental procedures were approved by the Institutional Review Board of Nanjing Medical University. For all of the cases, the pathological diagnosis and grading were confirmed by two experienced pathologists in accordance to the principles of World Health Organization Classification. Types of tissues used are listed in Table I and II.

### Tissue microarray and immunohistochemistry

Two separated cores taken from paraffin-embedded specimens of 90 glioma cases and 9 normal brain tissues were used to construct tissue microarrays. Slides and sections of the glioma tissues were stained with GSK-3β (1:50 dilution) and phospho-GSK-3β (Ser9) (1:100 dilution) antibodies (Cell Signaling, MA, USA), and then detected by EnVision DAB kit from Dako (Carpinteria, CA, USA). Staining intensity levels were independently scored by two observers. Average staining scores were determined for tissue cores of each case. Histopathologic diagnosis was examined independently by immunohistochemical analysis to reduce bias in scoring staining intensity. Staining intensity is graded on a 0–3 scales, on which 0 indicated no staining, 1 indicated weak, 2 indicated moderate, 3 indicated the most intense.

### Adenovirus preparation and transduction efficiency

The GSK-3β cDNA was sub-cloned into pAdtrack-CMV shuttle vector. The resulting plasmid pAdtrack-CMV-GSK-3β was linearized with Pme I followed by homologous recombination with backbone plasmid pAdEasy-1 in BJ5183 cells to generate recombinant adenovirus plasmid pAd-GSK-3β (Ad-GSK-3β). The DNA of identified Ad-GSK-3β was digested with Pac I, and transfected into 293A cells by lipofectamine-mediated method to package recombinant adenovirus. The titre of the Ad-GSK-3β was measured with the aid of green fluorescence protein (GFP) expression after multiplication and purification. In general, the cell infection was dose-dependent in the range of 5–20 MOI. More than 80% of infected cells were obtained at 10 MOI for U87 and U251 cells after 72 h; thus cells were infected at 10 MOI from the studies as described in Figures 2–4.

### Immunoblotting

Tissues and cells were lysed on ice for 30 min in RIPA buffer (100 mM Tris, 150 mM NaCl, 1% Triton, 1% deoxycholic acid, 0.1% SDS, 1 mM EDTA, and 2 mM NaF) supplemented with 1 mM sodium vanadate, 2 mM leupeptin, 2 mM aprotinin, 1 mM phenylmethylsulfonyl fluoride (PMSF), 1 mM DTT, and 2 mM pepstatin A. The supernatant was collected after centrifugation at 12,000 *rpm/min* for 15 min, and protein concentration was determined using protein assay reagent from Bio-Rad (Hercules, CA, USA). Proteins were electrophoretically separated by SDS-PAGE and transferred to nitrocellulose (Trans-Blot Transfer Medium, Bio-Rad). Membranes were blocked with 5% nonfat dry milk in PBS containing 0.05% Tween 20, and incubated with specific antibodies. Protein bands were detected by incubation with horse radish peroxidase-conjugated antibodies (Cell signaling), and visualized through enhanced chemiluminescence reagent (Thermo Fisher).

### Reverse transcription–PCR (RT–PCR)

Total RNAs were extracted using Trizol reagent (Invitrogen, Carlsbad, CA, USA) according to the manufacturer's instruction. Two micrograms of total RNAs were used for cDNA synthesis using PrimeScript RT Master kit (TaKaRa, Dalian, China). Reverse transcription-PCR (RT-PCR) analysis was performed using the cDNAs as the template. PCR was amplified for less than 30 cycles. Levels of glyceraldehyde-3-phosphate dehydrogenase (GAPDH) were used as the internal control. Primers for the PCR analysis were as follows: VEGF, 5′-TCGGGCCTCCGAAACCATGA-3′ (sense), and 5′-CCTGGTGAGAGATCTGGTTC-3′ (antisense); and GAPDH, 5′-CCACCCATGGCAAATTCCATGGCA-3′ (sense) and 5′-TCTAGACGGCAGGTCAGGTCCACC-3′ (antisense).

### ELISA assay

Capture ELISA was performed using a human VEGF ELISA kit according to the manufacturer's instruction (R&D Systems). Optical densities were read at 405 nm, and the rate of VEGF secretion was calculated as previously described [19].

### Angiogenesis and tumor growth assay

The 4-week-old male nude mice [BALB/cA-nu (nu/nu)] were purchased from Shanghai Experimental Animal Center (Chinese Academy of Sciences, China), maintained in pathogen-free conditions, and sustained with standard diets. All studies were approved by the Institutional Committee on Animal Care of Nanjing Medical University. For angiogenesis assay in nude male mice, animals were randomly divided into two groups (*n* = 5). U87 cells were trypsinized, and suspended in serum-free medium (5 × 10^7^ cells/ml). Aliquots of cells (5 × 10^6^ cells) were mixed with 0.2 ml growth factor-reduced phenolred-free Matrigel (BD Biosciences, MA, USA), and injected subcutaneously into both flanks of BALB/cA-nude mice. The Matrigel plugs were removed from the mice 9 days after the implantation, and surrounding tissues were trimmed. The plugs were photographed and weighed, and immersed immediately into lysis buffer (1 mM EDTA and 5 mM phosphate, pH 8), and incubated at 4°C for 24 h. Hemoglobin content levels were measured using a Drabkin's reagent kit (Sigma-Aldrich, St Louis, MO) based on the manufacturer's instruction.

For tumor growth assay, animals were randomly divided into two groups (*n* = 8). A total of 1 × 10^7^ U87 cells suspended in serum-free DMEM medium were transplanted subcutaneously into both flanks of nude mice. The mice were euthanized 30 days after the injection, the xenograft tumors were removed from the mice and analyzed. Tumor tissues were embedded in paraffin, cut into 5 μm, and placed sections onto glass slides. Sections of tumors were stained with monoclonal antibody against CD31, and detected through streptavidin-biotin-horse radish peroxidase complex (SABC) formation. The relative angiogenesis levels were estimated by micro-vessel density (MVD) as previously described [20].

### Statistical analysis

Statistical analysis was performed using Stata statistical package (Version 10.0, Stata Corp, LP, USA). Pearson χ^2^ test or Fisher exact tests were used to compare qualitative variables, and the differences are considered significance at *P* < 0.05. The quantitative variables were analyzed by the Student *t*-test.
